# Silk Composite with a Fluoropolymer as a Water-Resistant Protein-Based Material

**DOI:** 10.3390/polym10040459

**Published:** 2018-04-21

**Authors:** Keiji Numata, Nao Ifuku, Akira Isogai

**Affiliations:** 1 Biomacromolecules Research Team, RIKEN Center for Sustainable Resource Science, 2-1 Hirosawa, Wakoshi, Saitama 351-0198, Japan; nao.ifuku@riken.jp; 2 Department of Biomaterial Sciences, Graduate School of Agricultural and Life Sciences, The University of Tokyo, 1-1-1 Yayoi, Bunkyo-ku, Tokyo 113-8657, Japan; aisogai@mail.ecc.u-tokyo.ac.jp

**Keywords:** silk proteins, fluoropolymer, contact angle, vapor barrier, shrinkage test, biodegradability

## Abstract

Silk-based materials are water-sensitive and show different physical properties at different humidities and under wet/dry conditions. To overcome the water sensitivity of silk-based materials, we developed a silk composite material with a fluoropolymer. Blending and coating the silk protein-based materials, such as films and textiles, with the fluoropolymer enhanced the surface hydrophobicity, water vapor barrier properties, and size stability during shrinkage tests. This material design with a protein biopolymer and a fluoropolymer is expected to broaden the applicability of protein-based materials.

## 1. Introduction

Spider and silkworm silk proteins (fibroins) have been investigated widely because of their toughness, light weight, biodegradability and biocompatibility [1,2,3,4]. In addition to silk fibers and textiles, silk protein is processable and has been used to generate various biopolymer-based materials, including nanoparticles, porous materials, films, sponges, and hydrogels, for use in biomedical applications such as tissue engineering, drug/gene delivery and regenerative medicine [5,6,7]. In nature, silk fiber is used as a structural material in spider webs, spider draglines and silkworm cocoons [8,9]. However, silk protein has not yet been used as a structural material on a bulk scale. One of the limitations and drawbacks to the practical use of silk as a structural material is its water sensitivity, namely, silk protein-based materials are highly sensitive to water.

Water molecules in silk-based materials are categorized roughly into two types, namely, bound water and free water. Bound water molecules strongly interact with silk molecules and show different characteristics to those of bulk water; in contrast, free water means unbound water molecules that behave similarly to bulk water [10,11,12,13]. The influences of bound water on the biological and physical properties of silk molecules have been widely reported by several groups. Asakura and coworkers reported that the hydration of *Bombyx mori* silk molecules induces the stabilization of silk I forms based on solid-state NMR analysis [14]. The secondary structure and dynamics of swollen *B. mori* silk molecules were also characterized by ^13^C- and ^1^H-pulsed NMR [15,16]. Asakura et al. have also reported that hydration does not affect the crystalline fraction of *B. mori* silk fibers [17]. It was reported that bound water influences the glass transition temperature (*T*_g_) of *B. mori* silk molecules, that is, the *T*_g_ decreases with an increase in the water content of the silk films [18,19,20]. In addition to *B. mori* silk, the storage modulus and loss tangent of *Nephila edulis* spider dragline [21,22] and the elastic modulus values of *Antheraea pernyi* silkworm silk [23] and *Argiope trifasciata* spider silk [24] are reported to be affected by bound water. Bound water is considered to disrupt the hydrogen bonds between silk molecules in amorphous phase, and hence to enhance the mobility of the silk molecules, as well as influence the glass transition behavior [18,19,20,21,25]. Considering the biological properties of silk-based materials, the state of the water molecules in a silk hydrogel controls the effect of the hydrogel on cell viability, namely, human cell lines and cell-adhesion proteins in the extracellular matrix preferentially expand and adhere on silk molecules hydrated with more bound water [13].

Macroscopic studies on the effect of water and relative humidity (RH) on silk materials have been reported by several groups. Cebe and coworkers reported that hot-water vapor annealing induces crystallization in silk films [26]. In our previous studies, we demonstrated the effects of the water content in silk films and fibers on crystallization, bio- and thermal degradation [27,28]. By using thermal gravimetric analysis and differential scanning calorimetry (DSC), the silk samples prepared at different RHs were analyzed in terms of the effects of the water content on thermal degradation, crystallization and transition of *B. mori* silk materials. The hydration state and RH affected the mechanical properties of silk fibers [23]. At a relatively high RH, approximately 97%, the toughness and degree of crystallinity of silk films increase dramatically, indicating that the appropriate hydration of silk molecules induces crystallization and plasticization simultaneously [28]. The RH from 20% to 60% resulted in tough and strong silk materials by using various types of silk hydrogels. Dehydration did not negatively impact the biodegradability of the silk resins and hydrogels [27]. Thus, the thermal stability, mechanical properties and other attributes of silk materials are regulated by their water content and crystallinity. To exploit silk and silk-based materials as practical structural materials for human use, stabilization against water molecules is necessary.

As introduced above, silk-based materials are water-sensitive and show different physical properties at different humidities. However, this instability of silk under wet conditions is detrimental to its use as a structural material. To overcome the water sensitivity of silk-based materials, in this study we developed a silk composite material with a fluoropolymer, which is famous for its hydrophobicity and waterproofness. Blend films of silk proteins and fluoropolymer showed enhanced surface hydrophobicity and vapor barrier properties. The coating of fluoropolymer on silk textiles was resistant to washing and shrinkage treatments. This material design, with a protein biopolymer and a fluoropolymer, will broaden the applicability of protein-based materials.

## 2. Materials and Methods

### 2.1. Preparation of Silk Powder 

To obtain silk powder samples, silk fibroin solution was prepared according to a previously reported method [7,29]. Briefly, *B. mori* silkworm cocoons were cut and boiled for 30 min in a 0.02 M Na_2_CO_3_ solution, and subsequently washed with MilliQ water to remove wax layers and sericin. The extracted silk fibroins were dried at 25 °C for 24 h, and dissolved in a 9.3 M LiBr solution at 60 °C for 2 h at a concentration of 200 g/L. The silk solution was dialyzed with MilliQ water for at least 4 days using a dialysis membrane (Pierce Snake Skin MWCO 3500; Thermo Fisher Scientific, Waltham, MA, USA). Dialysis was completed when the conductivity of the dialysis solution was identical to that of MilliQ water. The silk solution was lyophilized to yield the silk powder.

### 2.2. Film Preparation

Silk powder was dissolved into hexafluoroisopropanol (HFIP) to generate a silk HFIP solution (25 g/L). It took approximately 24 h to dissolve the silk powder in the HFIP at 25 °C. The silk HFIP solution was blended with a fluoropolymer, Lumiflon^®^ LF600X (AGC Chemical Company, Tokyo, Japan). The solvents (xylene and ethylbenzene) in the Lumiflon^®^ LF600X solution (50 wt % fluoropolymer, 26 wt % xylene, 24 wt % ethylbenzene) were evaporated, and the resulting solid fluoropolymer was dissolved into HFIP (25 g/L). To prepare the film samples, the silk HFIP solution and the fluoropolymer HFIP solution were mixed and casted on a Teflon Petri dish. After drying for 16 h, films with a thickness of approximately 50 μm were obtained.

### 2.3. Tensile Tests

The tensile tests of the film samples were performed by a mechanical testing apparatus (EZ-LX/TRAPEZIUM X, Shimadzu, Kyoto, Japan) [30]. The initial length of the film sample was approximately 5 mm. The extension speed was 10 mm/min, and a 500 N load cell was used. The strength at break, Young’s modulus, elongation at break, and toughness were obtained based on the resultant stress–strain curves.

### 2.4. Thermal Analysis

DSC measurements were performed using a DSC 8500 (Perkin Elmer Inc. Waltham, MA, USA) to quantitatively characterize the thermal properties of the silk and fluoropolymer according to a previous report [30]. Approximately 10 mg of the sample was transferred to a DSC aluminum pan which was first cooled to −60 °C, and then heated to 240 °C at a rate of 20 °C/min. The glass transition (*T*_g_) and water evaporation were determined from the DSC thermograms.

### 2.5. Scanning Electron Microscopy (SEM) Observations 

The sample morphology was analyzed by SEM. Silk samples were sliced into small pieces approximately 2 mm × 2 mm × 1 mm. The sliced samples were mounted onto an aluminum stub, sputter-coated with gold, and imaged by SEM (JSM6330F, JEOL Ltd., Tokyo, Japan) at an accelerating voltage of 5 kV. The cross-section of the samples was prepared using a microtome (RM2265, Leica Microsystems GmbH, Wetzlar, Germany) with a diamond blade.

### 2.6. Advancing Contact Angle of Water

The wettability of the cast film surface was estimated by the advancing contact angle (*θ*_adv_) measurement with distilled water, using a FACE Contact Angle Meter CA-X (Kyowa Interface Science, Saitama, Japan), according to a previous procedure [31]. Cast films of the samples were prepared on glass substrates by solution casting from the silk and fluoropolymer HFIP solutions, as described above. The *θ*_adv_ value was calculated as the average of ten data obtained at different points on the surface (*n* = 5).

### 2.7. Water Vapor Barrier Test

The water vapor permeability rates of the film samples were determined at 37 °C using a Mocon Permatran-W model 1/50G (Modern Controls, Minneapolis, MN, USA) under standard conditions (ASTM 3985) [32]. Each measurement was continued until the water vapor permeability rate reached a stable value.

### 2.8. Shrinkage Test

*B. mori* silk textiles were kindly provided by Spiber Inc. (Tsuruoka, Japan). The silk textiles were immersed in Lumiflon^®^ LF600X (50 wt % fluoropolymer, 26 wt % xylene, 24 wt % ethylbenzene) for 1 min, and then dried in air at 25 °C and an RH of approximately 40%. Square silk textile samples (approximately 50 mm × 50 mm) with and without a fluoropolymer coating were used for the shrinkage test. The samples were immersed in hot water (40 °C) for 10 min and were dried at 25 °C and an RH of 65% for 16 h. This washing and drying cycle was performed three times. To evaluate the shrinkage of the samples, the squares of the samples were measured, and the changes in the area of the samples were determined. The test was performed three times, and the results are expressed as mean values and standard deviations.

### 2.9. Biodegradation Test

The biochemical oxygen demand (BOD) test was performed to determine the biodegradability of the silk samples in activated sludge (Chemicals Evaluation and Research Institute, Tokyo, Japan) with an Oxitop IS-6 (WTW GmbH, Weilheim in Oberbayern, Germany), according to a previous procedure [33]. A sample film (approximately 10 mg) was immersed in 100 mL of activated sludge at 25 °C for 30 days. The activated sludge was replaced with fresh sludge every 5 days. Before and after the biodegradation test, the morphologies and mechanical properties of the samples were characterized by the methods explained above.

## 3. Results and Discussion

### 3.1. Basic Properties of Silk/Fluoropolymer Blend Films

**Figure 1:**
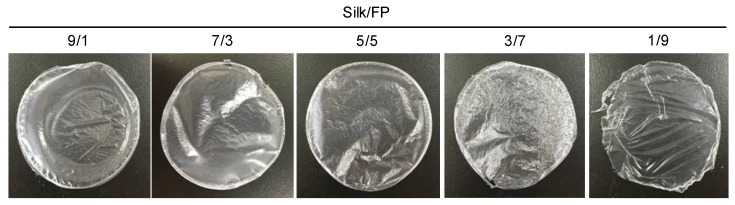
Silk/fluoropolymer (FP) blend films prepared at different silk/FP blending weight ratios.

**Figure 2:**
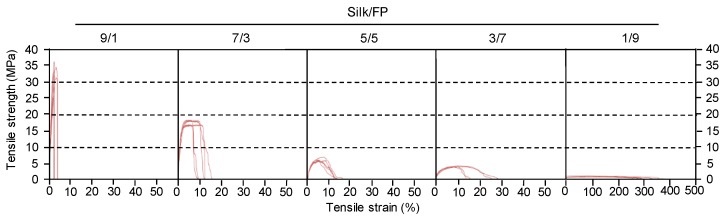
Typical stress-strain curves of silk/fluoropolymer blend films prepared at different blend ratios. Five curves are shown for each ratio.

Blend films of silk and fluoropolymer were prepared at different blend ratios (wt %) (Figure 1). All the films were transparent, but the films with lower silk contents such as silk/fluoropolymer (FP) = 1/9 were too elastic to be handled. The mechanical properties of the silk/FP blend films were characterized in terms of their stress–strain curves (Figure 2). With an increase in the fluoropolymer content, the blend films became more elastic and stretchable.

**Figure 3:**
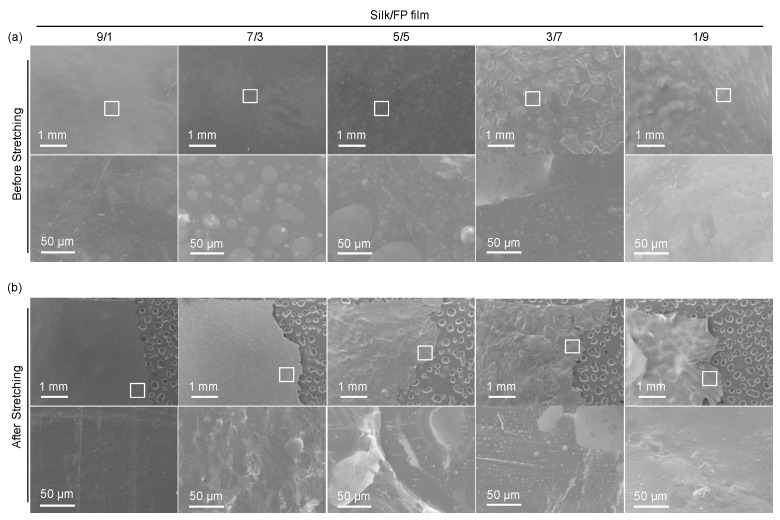
SEM images of the silk/fluoropolymer (FP) blend films before (**a**) and after the stretching mechanical test (**b**). Bottom images in (**a**) and (**b**) are the enlarged images of the areas indicated by the squares in the top images.

The surface morphologies of the film samples were observed by SEM (Figure 3a). We could recognize the domain structures at the surfaces. In the enlarged images of the 7/3 and 5/5 silk/FP blend films, the fluoropolymer domains were obvious. In the cases of the 9/1 and 1/9 silk/FP blend films, minor domains were obscure but detectable. After stretching the film samples, we imaged the film surfaces close to the fracture edges (Figure 3b). The round domains were stretched and became more crack-like, indicating that silk/FP domains have a substantial impact on mechanical properties. Furthermore, silk and the fluoropolymer were not perfectly miscible at blend ratios between 9/1 and 1/9.

**Figure 4:**
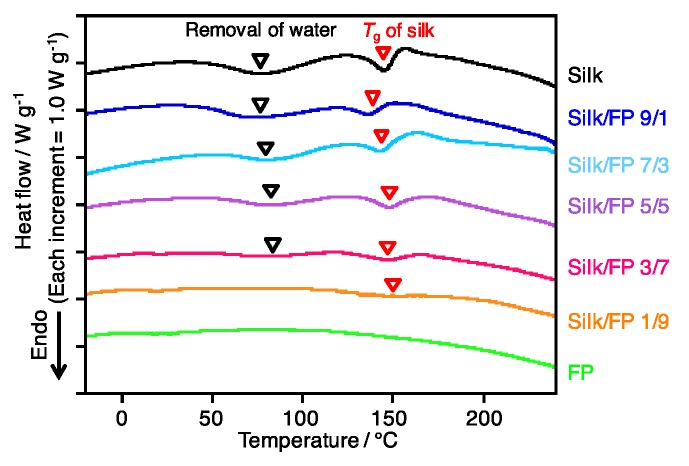
DSC profiles of silk, fluoropolymer (FP) and silk/FP blend films from −60 to 240 °C at 20 °C/min.

To clarify the blending state, namely, to determine the miscibility of silk and fluoropolymer, the blend films were characterized by DSC. The peaks originating from the removal of water, and the *T*_g_ of silk were assigned as shown in Figure 4. On the other hand, the fluoropolymer did not show clear peaks or transitions. In terms of the shift in the silk *T*_g_, silk domains were present at silk/FP ratios from 9/1 to 1/9, and their *T*_g_ values were not significantly influenced by the fluoropolymer. This result indicates that the silk and fluoropolymer were not miscible in the blend films, which was confirmed by the SEM micrographs shown in Figure 3.

**Figure 5:**
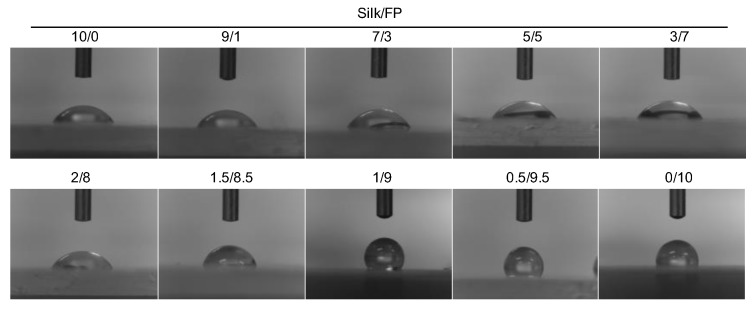
Contact angles of silk, fluoropolymer (FP) and silk/FP blend films. The 10/0 and 0/10 images are those of the silk film and fluoropolymer film, respectively.

**Figure 6:**
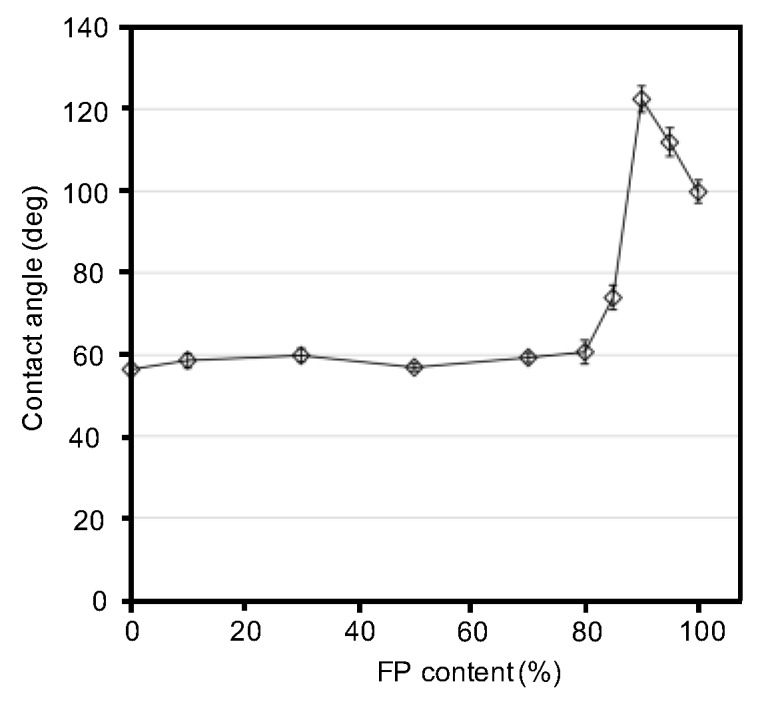
Contact angles of the silk, fluoropolymer (FP) and silk/FP blend films, as a function of fluoropolymer content. The 10/0 and 0/10 values are those of the silk film and fluoropolymer film, respectively.

The hydrophobicities of the blend films were characterized based on their advancing contact angles (Figure 5). Based on the contact angles of water droplets on the blend films, we clarified the trend in the hydrophobicity of the blend films as a function of fluoropolymer content (Figure 6). As expected, the contact angle increased with an increase in fluoropolymer content. However, surprisingly, the blend film (silk/FP = 1/9) showed the highest contact angle, approximately 120°, which might be because the perfluoro groups of the fluoropolymer were aligned on the surface by the presence of the silk molecules.

**Figure 7:**
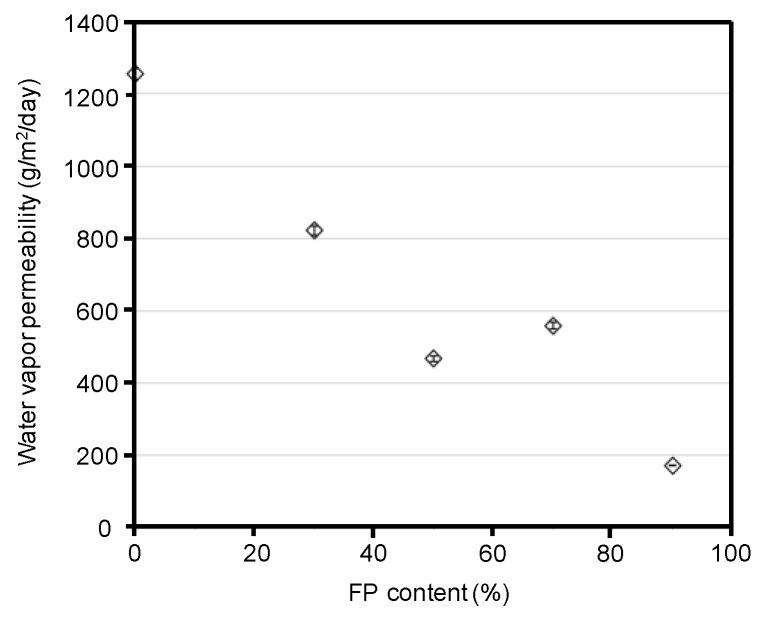
Water vapor permeability of silk and silk/fluoropolymer (FP) blend films as a function of fluoropolymer content. Zero fluoropolymer content represents the silk film.

In addition to the hydrophobicities of the blend films, we evaluated their water vapor barrier properties (Figure 7). The water vapor barrier properties are related to not only the surface morphologies, but also the interior structures of the blend films. The water vapor permeability decreased with an increase in fluoropolymer content. The silk/FP blend film (1/9) showed a water vapor permeability of approximately 170 g/m^2^/day. Although nylon has a similar polyamide structure to that of proteins, in that both contain amide bonds; the water vapor permeability of nylon 6 and nylon 6, 10 are 47 and 22 g/m^2^/day, respectively. For practical uses of silk materials, we expect that the water vapor permeability of silk blend materials will be approximately 100 g/m^2^/day. However, the blend film did not show such a low water vapor permeability in this study.

### 3.2. Fluoropolymer Coating with Silk Materials

To enhance the water resistance of silk materials, we evaluated the fluoropolymer coating of the silk materials. *B. mori* silk textile and silk film were coated with the fluoropolymer. The coating of the fluoropolymer on the silk films was approximately 50 µm thick, based on the cross-section SEM observation of the fluoropolymer-coated silk films. The fluoropolymer-coated silk films show a contact angle of approximately 90°. The water vapor permeability of the fluoropolymer-coated silk films was approximately 110 g/m^2^/day. These properties of the fluoropolymer-coated silk films indicate the potential advantages of a fluoropolymer coating on silk materials.

**Figure 8:**
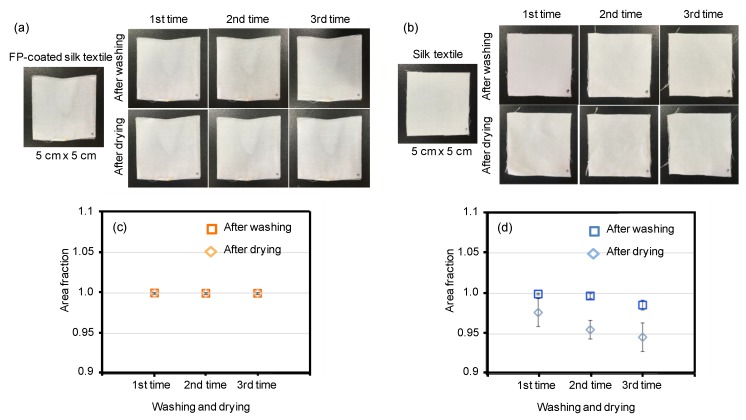
Shrinkage tests of silk textiles with and without a fluoropolymer (FP) coating. Pictures of fluoropolymer-coated silk textile (**a**) and mock silk textile (**b**) before and during the washing/drying cycles. The area changes of the samples during the washing and drying processes. The original area was 50 mm × 50 mm. Each data point is expressed as the mean and standard deviation.

To show the excellent water resistance of the fluoropolymer-coated silk-based materials, shrinkage tests, which were similar to the washing and drying process used for clothing, were performed with the silk textiles. A shrinkage test was performed on the silk textile, but not on the silk film, as the shrinkage test is designed for clothes. We coated *B. mori* silk textile with fluoropolymer and subjected it to the shrinkage test (Figure 8). The coated textile showed great size stability during the washing/drying cycles (Figure 8a,c). In contrast, the silk textile without the fluoropolymer coating did not show size stability (Figure 8b). After the third drying stage, the sizes had decreased by approximately 5% (Figure 8d). Thus, the fluoropolymer coating can enhance the water resistance of silk materials in practical applications.

### 3.3. Biodegradability of Silk, Fluoropolymer-Coated Silk, and Silk/Fluoropolymer Blend

**Figure 9:**
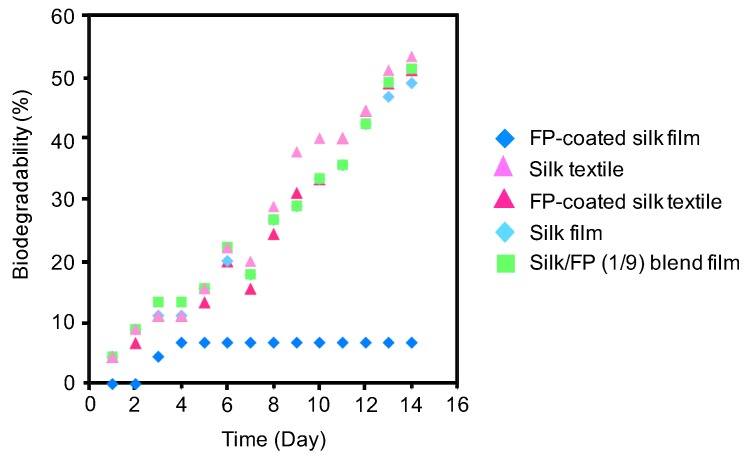
Biochemical oxygen demand (BOD) curves of the silk film, fluoropolymer (FP)-coated silk film, silk textile, FP-coated silk textile, and silk/FP (1/9) blend film in active sludge at 25 °C.

To study the effect of the fluoropolymer on the biodegradability of silk materials, a BOD test was performed using the silk film, fluoropolymer-coated silk film, silk textile, fluoropolymer-coated silk textile, and silk/FP (1/9) blend film in active sludge at 25 °C (Figure 9). All the silk materials showed increases in BOD with increases in degradation time, except for the fluoropolymer-coated silk film. The fluoropolymer-coated silk film was not significantly degraded after being subjected to active sludge for 14 days, indicating that the fluoropolymer coating prevents biological degradation of silk films. In contrast, the fluoropolymer coating on the silk textile did not prevent biological degradation.

**Figure 10:**
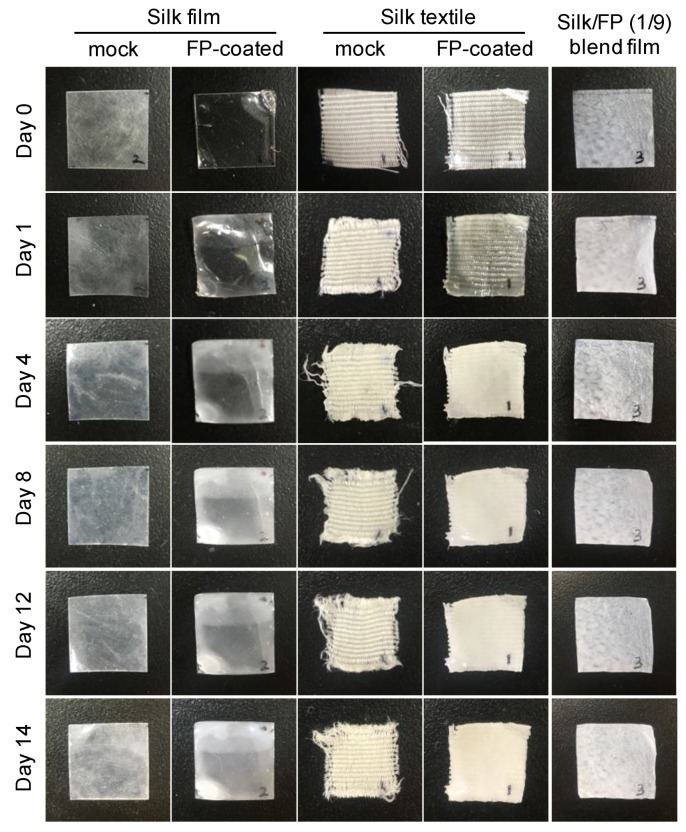
Pictures of silk materials before and after the biodegradation test in active sludge at 25 °C.

Before and during the biodegradation tests, the sample morphologies noticeably changed (Figure 10). The silk film without the fluoropolymer coating became white during the biodegradation test, indicating that the surface of the silk film degraded and became rough. The fluoropolymer-coated silk film also became white and opaque, even though the surface was coated with fluoropolymer. The silk textile samples with and without the fluoropolymer coating gradually changed, especially at the edges of the samples. The silk/FP (1/9) blend film became white and opaque.

**Figure 11:**
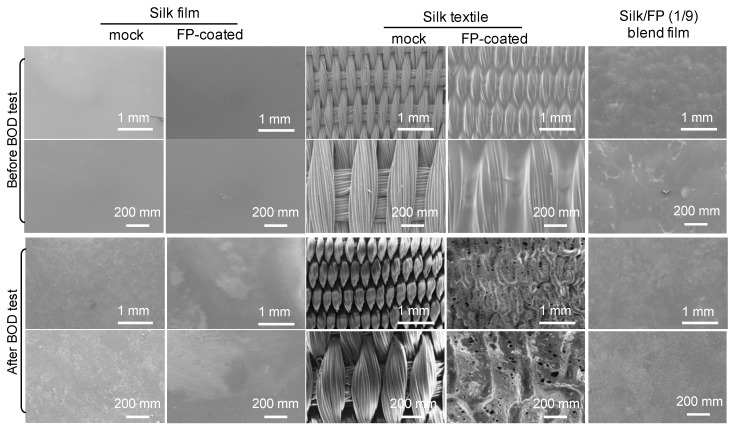
SEM images of silk film (mock), fluoropolymer (FP)-coated silk film, silk textile, FP-coated silk textile, and silk/FP (1/9) blend film before and after BOD test in active sludge at 25 °C for 14 days. The bottom image for each condition is an enlarged section of the top image.

To visualize the details of the surface morphologies, as well as to clarify the difference in the degradability of silk film and textiles, we observed the silk samples by SEM before and after the biodegradation test (Figure 11). The surface of the mock silk film without any coating was degraded and roughed by the biodegradation treatment, while the FP-coated silk film was not noticeably degraded. Based on the BOD tests (Figure 9), silk textile samples, even those with a fluoropolymer coating, were degraded in active sludge. The SEM images of the fluoropolymer-coated silk textile showed degradation at the surface (FP-coated silk textile after BOD test shown in Figure 11). Thus, the simple dipping of the silk textile in the fluoropolymer solution was not sufficient to perfectly coat the silk textile and protect it against biological degradation. The silk/FP blend film showed a rough surface morphology after the BOD test, indicating that the blend film was also degraded in the active sludge.

**Figure 12:**
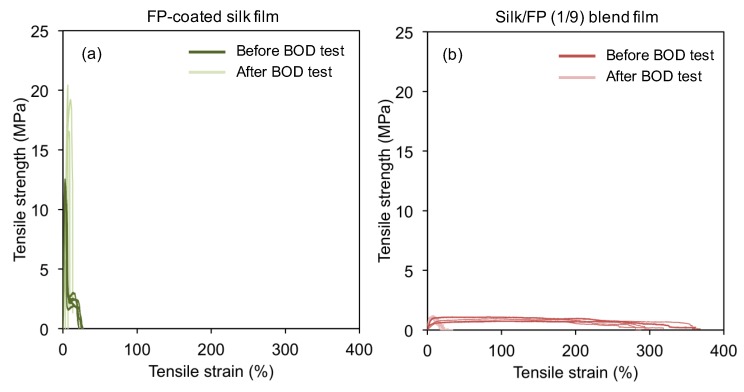
Typical stress-strain curves of the fluoropolymer (FP)-coated silk film (**a**) and the silk/FP (1/9) blend film (**b**) before and after the BOD test. Five curves are shown in each graph.

To confirm the biodegradation-resistance of the fluoropolymer-coated silk films, we further characterized their mechanical properties before and after the BOD test. After the treatment with active sludge for 14 days, the silk films were too brittle for the mechanical tests. Therefore, we could not measure the stress-strain curves of the silk films after the BOD test. The fluoropolymer-coated silk films were not dramatically changed by the biodegradation test in terms of their mechanical properties (Figure 12a). On the other hand, the silk/FP (1/9) blend film was weakened by biodegradation (Figure 12b). Thus, the combination of the BOD test and mechanical analysis of the silk materials confirmed that fluoropolymer coating can prevent biodegradation of silk films, even in active sludge.

## 4. Conclusions

In this study, we developed a silk composite material with a fluoropolymer to overcome the water sensitivity of silk-based materials. Blending and coating silk protein-based materials, such as films and textiles, with a fluoropolymer enhanced the surface hydrophobicity, water vapor barrier properties, and size stability during washing/drying cycles. However, the blending of the fluoropolymer with silk proteins cannot stabilize the silk materials perfectly. In the case of the silk films coated with the fluoropolymer, the water and biodegradation resistances of the silk materials were improved. Thus, fluoropolymer treatment is expected to broaden the applicability of silk materials as well as protein-based materials. The other technique to modify the surface property of silk materials is plasma treatment. However, plasma treatment sometimes digests silk molecules at the surface of the silk materials, resulting in more water-sensitive surface. The fluoropolymer coating also has a disadvantage, namely, the loss of the original texture of the silk materials. To resolve this issue, we plan to design and synthesize other fluoropolymers to realize a fluoro-coating layer with a thickness of molecular level. The combination of protein-based materials and fluoropolymers will open a door for many applications in biomaterial, structural material and apparel material fields.

## Abbreviations

The following abbreviations are used in this manuscript:

RH	relative humidity
DSC	differential scanning calorimetry
NMR	nuclear magnetic resonance
HFIP	hexafluoro-2-propanol
SEM	scanning electron microscopy
*θ*_adv_	advancing contact angle
FP	fluoropolymer
*T*_g_	glass transition temperature
BOD	biochemical oxygen demand

## References

[1] Kaplan D.L., Mello C.M., Arcidiacono S., Fossey S., Senecal K., Muller W. (1997). Silk. Protein-Based Materials.

[2] Pritchard E.M., Kaplan D.L. (2011). Silk fibroin biomaterials for controlled release drug delivery. Expert Opin. Drug Deliv..

[3] Numata K., Cebe P., Kaplan D.L. (2010). Mechanism of enzymatic degradation of beta-sheet crystals. Biomaterials.

[4] Vollrath F., Porter D. (2006). Spider silk as a model biomaterial. Appl. Phys. A Mater..

[5] Altman G.H., Diaz F., Jakuba C., Calabro T., Horan R.L., Chen J., Lu H., Richmond J., Kaplan D.L. (2003). Silk-based biomaterials. Biomaterials.

[6] Numata K., Kaplan D.L. (2010). Silk-based delivery systems of bioactive molecules. Adv. Drug Deliv. Rev..

[7] Rockwood D.N., Preda R.C., Yucel T., Wang X., Lovett M.L., Kaplan D.L. (2011). Materials fabrication from bombyx mori silk fibroin. Nat. Protoc..

[8] Numata K. (2015). Poly(amino acid)s/polypeptides as potential functional and structural materials. Polym. J..

[9] Numata K., Masunaga H., Hikima T., Sasaki S., Sekiyama K., Takata M. (2015). Use of extension-deformation-based crystallisation of silk fibres to differentiate their functions in nature. Soft Matter.

[10] Kim Y.S., Dong L., Hickner M.A., Glass T.E., Webb V., McGrath J.E. (2003). State of water in disulfonated poly(arylene ether sulfone) copolymers and a perfluorosulfonic acid copolymer (nafion) and its effect on physical and electrochemical properties. Macromolecules.

[11] Kuntz I.D. (1971). Hydration of macromolecules. III. Hydration of polypeptides. J. Am. Chem. Soc..

[12] Lee K.Y., Ha W.S. (1999). DSC studies on bound water in silk fibroin/s-carboxymethyl kerateine blend films. Polymer.

[13] Numata K., Katashima T., Sakai T. (2011). State of water, molecular structure, and cytotoxicity of silk hydrogels. Biomacromolecules.

[14] Ishida M., Asakura T., Yokoi M., Saito H. (1990). Solvent-induced and mechanical-treatment-induced conformational transition of silk fibroins studied by high-resolution solid-state c-13 NMR-spectroscopy. Macromolecules.

[15] Asakura T., Demura M., Watanabe Y., Sato K. (1992). H-1 pulsed nmr-study of bombyx-mori silk fibroin—Dynamics of fibroin and of absorbed water. J. Polym. Sci. Polym. Phys..

[16] Yoshimizu H., Asakura T. (1990). The structure of bombyx-mori silk fibroin membrane swollen by water studied with ESR, C-13-NMR, and FT-IR spectroscopies. J. Appl. Polym. Sci..

[17] Asakura T., Isobe K., Aoki A., Kametani S. Macromolecules.

[18] Hu X., Kaplan D., Cebe P. (2007). Effect of water on the thermal properties of silk fibroin. Thermochim. Acta.

[19] Hu X., Kaplan D., Cebe P. (2008). Dynamic protein−water relationships during β-sheet formation. Macromolecules.

[20] Mo C., Wu P., Chen X., Shao Z. (2009). The effect of water on the conformation transition of bombyx mori silk fibroin. Vib. Spectrosc..

[21] Guan J., Porter D., Vollrath F. (2013). Thermally induced changes in dynamic mechanical properties of native silks. Biomacromolecules.

[22] Yuan Q., Yao J., Huang L., Chen X., Shao Z. (2010). Correlation between structural and dynamic mechanical transitions of regenerated silk fibroin. Polymer.

[23] Fu C., Porter D., Shao Z. (2009). Moisture effects on antheraea pernyi silk’s mechanical property. Macromolecules.

[24] Plaza G.R., Guinea G.V., Pérez-Rigueiro J., Elices M. (2006). Thermo-hygro-mechanical behavior of spider dragline silk: Glassy and rubbery states. J. Polym. Sci. Part B Polym. Phys..

[25] Agarwal N., Hoagland D.A., Farris R.J. (1997). Effect of moisture absorption on the thermal properties of bombyx mori silk fibroin films. J. Appl. Polym. Sci..

[26] Hu X., Shmelev K., Sun L., Gil E.S., Park S.H., Cebe P., Kaplan D.L. (2011). Regulation of silk material structure by temperature-controlled water vapor annealing. Biomacromolecules.

[27] Numata K., Ifuku N., Masunaga H., Hikima T., Sakai T. (2017). Silk resin with hydrated dual chemical-physical cross-links achieves high strength and toughness. Biomacromolecules.

[28] Yazawa K., Ishida K., Masunaga H., Hikima T., Numata K. (2016). Influence of water content on the beta-sheet formation, thermal stability, water removal, and mechanical properties of silk materials. Biomacromolecules.

[29] Jin H.J., Kaplan D.L. (2003). Mechanism of silk processing in insects and spiders. Nature.

[30] Malay A.D., Sato R., Yazawa K., Watanabe H., Ifuku N., Masunaga H., Hikima T., Guan J., Mandal B.B., Damrongsakkul S. (2016). Relationships between physical properties and sequence in silkworm silks. Sci. Rep..

[31] Numata K., Srivastava R.K., Finne-Wistrand A., Albertsson A.-C., Doi Y., Abe H. (2007). Branched poly(lactide) synthesized by enzymatic polymerization: Effects of molecular branches and stereochernistry on enzymatic degradation and alkaline hydrolysis. Biomacromolecules.

[32] Yang Q., Fukuzumi H., Saito T., Isogai A., Zhang L. (2011). Transparent cellulose films with high gas barrier properties fabricated from aqueous alkali/urea solutions. Biomacromolecules.

[33] Tachibana K., Urano Y., Numata K. (2013). Biodegradability of nylon 4 film in a marine environment. Polym. Degrad. Stab..

